# Going Beyond the Visible in Type 2 Diabetes Mellitus: Defense Mechanisms and Their Associations With Depression and Health-Related Quality of Life

**DOI:** 10.3389/fpsyg.2020.00267

**Published:** 2020-02-26

**Authors:** Gabriella Martino, Andrea Caputo, Federica Bellone, Maria C. Quattropani, Carmelo M. Vicario

**Affiliations:** ^1^Department of Clinical and Experimental Medicine, University of Messina, Messina, Italy; ^2^Department of Dynamic and Clinical Psychology, Sapienza University of Rome, Rome, Italy; ^3^Department of Cognitive Sciences, Psychology, Education and Cultural Studies, University of Messina, Messina, Italy

**Keywords:** defense mechanisms, depression, quality of life, physical component summary, mental component summary, chronic diseases, type 2 diabetes mellitus

## Abstract

**Introduction:**

Clinical psychological features may impact a person’s aptitude to deal with chronic diseases, leading to emotional distress, suffering, and a worse perceived quality of life (QoL). Chronic diseases are largely represented, and their incidence is constantly increasing all over the world. Type 2 Diabetes Mellitus (T2DM) is one of the most common chronic diseases and it is very difficult to manage, demanding long term self-management, which improves the perceived QoL. The aim of this study was to explore defense mechanisms, depression, QoL, time since diagnosis, and metabolic control in T2DM patients.

**Methods:**

51 patients with T2DM were assessed through a psychodiagnostic battery: Beck Depression Inventory-II, the 36-Item Short Form Health Survey, including indexes of Physical and Mental Component Summary and the Defense Mechanisms Inventory. Times since DM diagnosis and glycated hemoglobin values were detected.

**Results:**

Participants were mainly female (62.74%), with a mean age of 66.1 years. T2M time since diagnosis was 11.77 years (SD = 7.1). Mild depression was detected (with an overall score between 13 and 19). *Projection* was significantly associated with higher depression and with lower physical well-being; *Principalization* was negatively associated with depression and positively with both physical and mental well-being. *Turning Against Self* correlated positively with physical well-being and negatively with mental well-being. *Reversal* was associated with lower depression and higher mental well-being. A negative high correlation emerged between depression and mental well-being. Finally, a significant relationship was found between Projection and higher time since diagnosis (*r* = 0.31, *p* < 0.05).

**Conclusion:**

The correlations between defense mechanisms, depression and health-related QoL highlight the potential personification and protagonization, which may increase over time due to the illness intrusiveness and worsening of diabetes symptoms. The positive association between defensive strategies and well-being measures should be cautiously considered.

## Introduction

Clinical psychological features may impact a person’s aptitude to deal with chronic diseases, which may lead to emotional distress, suffering, and a worse perceived quality of life (QoL). Chronic diseases are largely represented among the general population, and their incidence is constantly increasing all over the world. It has been recognized that affective and emotional symptoms, such as depression and anxiety, respectively, could embody predictors of several chronic diseases (Conversano et al., 2015; Catalano et al., 2017, 2018; Martino et al., 2018a, b, 2019b,a; Kelly et al., 2019; Marchi et al., 2019). Particularly, psychological factors may compromise patients’ compliance and adherence, and this could increase mortality and mobility independently from many confounders (Wang et al., 2016). It is known that diabetes is one of the most common chronic diseases in the world and it is very difficult to manage, demanding long term self-management through constant blood glucose monitoring, a balanced diet, physical exercises and medical treatment, which improve perceived QoL (Conversano et al., 2009; Veltri et al., 2012; Palagini et al., 2016; Marchini et al., 2018; Catalano et al., 2019b; Conversano, 2019; Guicciardi et al., 2019; Martino et al., 2019d, c; Merlo, 2019; Quattropani et al., 2019; Lenzo et al., 2020). In fact, both adequate compliance and adherence prevent its relative outcomes leading to a lower risk of developing related complications (American Diabetes Association, 2018). Due to this, self-care could be considered as a suggestive index of patients’ adaptation to diabetes, which may involve psychological adjustment to disease and its related emotional distress (Lapolla et al., 2012; Schmitt et al., 2014; Del Piccolo et al., 2015; Craparo et al., 2016; Settineri et al., 2019a, b; Knowles et al., 2020). Emotional distress could exist independently from such chronic disease, though considering the higher risk of mortality and morbidity due to diabetes, a psychological elaboration processing would be useful for the psychic integration of the chronic illness experience within patients’ daily life (Whittemore et al., 2010; Castelnuovo et al., 2015; Van Houtum et al., 2015; Stanton and Hoyt, 2017; Di Giuseppe et al., 2018, 2019; Savarese et al., 2018; Catalano et al., 2019a). Demographical and physical characteristics, such as body mass index (BMI) and smoking and alcohol consumption, could predict depression in Type 2 Diabetes Mellitus (T2DM) (Bouwman et al., 2010; Rosa et al., 2019). Smith et al. (2013) observed a significant correlation between diabetes, anxiety symptoms and anxiety disorders, and highlighted that a fruitful psychological adjustment to diabetes is significantly associated with a better metabolic control, self-care and higher QoL. Moreover, controversial data support T2DM as a relevant risk factor for anxiety and highlight that good health, in the absence of T2DM, could represent a protective factor for anxiety. Martino et al. (2019b) underlined the predictive role of anxiety levels and disease duration in health related QoL in patients living with T2DM. Particularly, they observed a significant impact of time since diagnosis on Physical Component Summary (PCS) and a higher significant impact of anxiety and depression levels on both PCS and Mental Component Summary (MCS). The role of negative emotions and illness distress is deemed relevant for both the course and treatment efficacy in patients with T2DM (Heianza et al., 2015). In this regard, it is well acknowledged that feelings of loss, guilt and anger are associated with both worse glycemic control and indicators of medical adherence, such as glycated hemoglobin levels (Whithorth et al., 2016). Therefore, the need for further clinical psychological research is advocated in order to explore less conscious strategies to handle such a chronic condition (Pouwer et al., 2010; Marchini et al., 2018).

In line with these pieces of research based on the fundamental role of personal adjustment to such a chronic illness related to both psychological and physical health status, the aim of this study was to explore the relationships between defense mechanisms and depression, perceived QoL, time since diagnosis, and metabolic control in patients with T2DM.

## Materials and Methods

### Participants

A convenience sample of 51 patients was recruited at the Outpatients Clinics of the Department of Clinical and Experimental Medicine, University Hospital of Messina, Italy. Enrolled patients had a certified diagnosis of T2DM, in agreement with the American Diabetes Association criteria (American Diabetes Association, 2018), and they satisfied inclusion and exclusion criteria before entering the study. Inclusion criteria were: age from 55 to 75 years old; time since diagnosis of T2DM >5 years; pharmacological treatment with hypoglycemic drug (Metformin) and adequate schedules in the last 12 months; a full screening for diabetic outcomes over the last 6 months; a Mini-Mental State Examination score (MMSE) >24, to ensure the full comprehension of the psychodiagnostic evaluation. The exclusion criteria were: neurologic or psychiatric condition or use of neuropsychotropic drugs; heart failure as New York Heart Association (NYHA) class >2; respiratory, kidney or liver failure higher than moderate; endocrine disorders other than DM, to avoid confounders; severe musculoskeletal diseases; cancer.

### Ethics Statement

The study was approved by the Institutional Ethical Committee of the University Hospital “G. Martino,” University of Messina, Italy. All participants were fully informed about the purpose of the research and gave their written informed consent, in accordance with the Declaration of Helsinki and its later amendments. Participants were evaluated by a researcher in clinical psychology and physicians. Data were analyzed anonymously.

## Measures

### Demographical and Clinical Data

Collected demographical data included: age, gender, education, smoking habits, and employment, considered as categorical variables. Medical information covered data on BMI, T2DM duration and related outcomes, and metabolic control.

### Clinical Psychological Evaluation

Clinical psychological assessment was performed through a gold standard psychological interview by a researcher in clinical psychology, in a confidential setting, to detect patients’ mental status (Fava et al., 2012; Langher et al., 2017). The gold standard interview was implemented with the administration of self-report tests, inventories and questionnaires.

Particularly, the Beck Depression Inventory - second edition (BDI-II) was administered to identify depressive symptoms. It consists of 21 items scored on a 3-points Likert scale from 0, not present, to 3, severe (Beck et al., 1996; Ghisi et al., 2006). In the present study, the reliability (Cronbach’s α) of the measure was 0.85.

The Italian version of the Short Form-36 (SF-36) (Ware and Sherbourne, 1992; Apolone and Mosconi, 1998) was used to measure patients’ perceived health-related QoL. SF-36 comprises eight domains, measuring perceived mental health, role emotional, social functioning, vitality, general health, bodily pain, role physical, physical functioning. This self-report questionnaire has a total score ranging from 0 to 100 points with lower scores suggesting a worse perceived QoL. It permits the appraisal of patients’ health status through two synthetic indexes, PCS and MCS, reflecting physical and mental well-being respectively. The scoring algorithm for PCS embraces physical functioning, role physical, bodily pain, general health, and vitality scales, while the scoring algorithm for MCS includes vitality, social functioning, role emotional, and emotional well-being scales. In the present study, the reliability (Cronbach’s α) of the measure was 0.69 and 0.79 for PCS and MCS respectively.

The Defense Mechanisms Inventory (DMI) (Gleser and Ihilevich, 1969; Ihilevich and Gleser, 1986, 1994) is a semi-projective questionnaire, which encourages the subject to identify him/herself with ten short semi-structured stories, detecting both the presence and uniformity of five defensive clusters. Particularly, it consists of two stories for each area of investigation, namely: authority, independence, masculinity or femininity, competition, and finally, the area of conflicts which arise in daily life experiences. Each story comprises 20 likely answers distributed into four groups. The first group embraces “items” investigating behavior in realistic situations, the second includes impulsive behavior, the third comprises thoughts, and the fourth group holds affects. The five defense styles explore: Turning Against Object (TAO), as aggression in terms of identification with the aggressor and displacement; Projection (PRO), as the justification of aggression against the considered object; Principalization (PRN), as isolation, rationalization and intellectualization; Turning Against Self (TAS), as aggression against the self in terms of masochism and introjection; and Reversal (REV), as repression, denial and reaction formation. In the present study, the reliability of the measure for each scale was as follows: TAO (α = 0.72), PRO (α = 0.69), PRN (α = 0.65), TAS (α = 0.71), REV (α = 0.70).

### Clinical Characteristics

Physical evaluation was performed, determining height, weight, and BMI, expressed as weight in kilograms divided by the square of height in meters (Kg/m^2^). The detection of glycated hemoglobin (HbA1c), expressed as per cent values (%), reveals the mean blood glucose concentration in the last 3 months offering fundamental data on patients’ metabolic control. T2DM pharmacological treatment and T2DM related outcomes, such as hypertension and atherosclerosis nephropathy, retinopathy, glaucoma, and neuropathy, were collected during the clinical interview and derived from patients’ medical records.

### Statistical Analysis

Statistical analysis was performed using IBM SPSS statistical version 25. Descriptive analyses were completed concerning the demographic variables and clinical psychological dimensions. Clinical psychological variables for DMI, BDI-II, PCS, and MCS were normally distributed. Pearson’s *r* correlations were performed to evaluate the relationships between defense mechanisms and both depression and perceived QoL. Moreover, the relationships between defense mechanisms, time since diagnosis and metabolic control were examined. *p* values <0.05 were considered as statistically significant.

## Results

The recruited 51 participants were mainly female (62.74%), with a mean age of 66.1 years (SD = 6.1), and a secondary or higher education in most cases (overall 77.84%). Concerning T2DM, on average, time since diagnosis was 11.77 years (SD = 7.1), and related complications were reported only in 30% of participants, who showed a good glycemic control overall. The clinical sample characteristics are reported in Table 1.

**TABLE 1:** Demographic and medical characteristics of the study sample.

Variable		*Clinical sample (n* = *51)*
Gender	M	37.25%
	F	62.74%
Education	Primary school	21.16%
	Secondary school	58.82%
	High school or higher degree	19.02%
T2DM time since diagnosis *(yrs)*		11.77 ± 7.1
HbA1c *(%)*		7.18 ± 0.9
T2DM related complications	Micro-vascular	15%
	Macro-vascular	15%
Age (*yrs)*		66.1 ± 6.1
BMI (*Kg/m^2^)*		29.9 ± 5.2
Current smoking		15%

Descriptive statistics of the study variables are shown in Table 2. Looking at the mean values of the defense mechanisms examined through DMI, compared to norms differentiated by gender of the Italian sample (Ihilevich and Gleser, 1994), Principalization was higher than one standard deviation in male participants (normative mean of 44.9 with a standard deviation of 7.8). Moreover, TAS and Reversal were found to be higher in both males (whose normative values were, respectively, *M* = 34.5, SD = 8.8 for TAS and *M* = 37.1, SD = 7.7 for REV) and females (whose normative values were, respectively, *M* = 33.5, SD = 8.1 for TAS and *M* = 30, SD = 7.6 for REV). According to the norms of the BDI-II in the Italian context (Ghisi et al., 2006), the present sample was characterized by mild depression (with an overall score between 13 and 19). With regard to health-related QoL, both PCS and MCS have values expressed in t-scores (*M* = 50, SD = 10) below one standard deviation from the mean, and lower than those of the Italian normative sample (Apolone and Mosconi, 1998).

**TABLE 2:** Descriptive statistic of the study variables.

Measure	Mean	SD
TAO	42.54	11.79
PRO	50.59	8.55
PRN	50.66	8.09
TAS	49.24	8.56
REV	59.93	11.28
BDI-II	15.15	8.96
PCS	37.68	9.41
MCS	39.32	12.29

*TAO, Turning against object; PRO, Projection; PRN, Principalization; TAS, Turning against self; REV, Reversal; BDI-II, Beck depression inventory-II; PCS, Physical component summary; MCS, Mental component summary.*

The interrelations among the study variables are shown in Table 3. With regard to defense mechanisms, some statistically significant associations were found, demonstrating an overall fall in medium size range. Specifically, Projection was associated with higher depression and with lower physical well-being, whereas, Principalization was negatively associated with depression and positively associated with both physical and mental well-being. TAS correlated positively with physical well-being and negatively with mental well-being. Further, Reversal was associated with lower depression and higher mental well-being. A negative high correlation also emerged between depression and mental well-being.

**TABLE 3:** Interrelations among the study variables.

	TAO	PRO	PRN	TAS	REV	BDI-II	PCS	MCS
TAO	–	–	–	–	–	–	–	–
PRO	0.40**	–	–	–	–	–	–	–
PRN	−0.70***	−0.62***	–	–	–	–	–	–
TAS	−0.41**	−0.32*	0.09	–	–	–	–	–
REV	−0.76***	−0.57***	0.55***	0.02	–	–	–	–
BDI-II	0.26	0.38*	−0.52***	0.30	−0.43**	–	–	–
PCS	–0.20	−0.34*	0.33*	0.33*	0.13	–0.17	–	–
MCS	–0.23	0.00	0.31*	−0.38*	0.37*	−0.59***	0.04	–

TAO, Turning against object; PRO, Projection; PRN, Principalization; TAS, Turning against self; REV, Reversal; BDI-II, Beck depression inventory-II; PCS, Physical component summary; MCS, Mental component summary. *statistically significant at *p* < 0.05, **statistically significant at *p* < 0.01, ***statistically significant at *p* < 0.001.

With regards to the associations between defense mechanisms and both time since diagnosis and metabolic control in patients with T2DM, the only statistically significant relationship was found between Projection and higher time since diagnosis (*r* = 0.31, *p* < 0.05).

## Discussion

It is acknowledged that T2DM may incite distress, as patients suffer with a need to self-manage such chronic disease, recurrently detect blood glucose levels, and show adequate compliance adherence (Marshall et al., 1997; Marchini et al., 2018), which facilitate the avoidance of unhealthy behavior and outcomes. It is also known that people with psychopathological features showed an amplified risk to develop T2DM onset at a 10-year follow-up, independently from conventional risk factors for DM (Engum, 2007; Shinkov et al., 2018).

Overall, compared to normal samples, the study participants seem to be characterized by a mild level of depressive symptoms, worse perceived QoL in both physical and mental terms, and a higher proneness to use some defense mechanisms, thus highlighting the underlying psychic suffering intertwined with T2DM.

With regards to the associations between defense mechanisms and both depression and health-related QoL, the present study highlights interesting findings in participants with T2DM. In more detail, Projection is found to be associated with higher depression, thus suggesting that depressive symptoms in such a sample are higher when there is a greater tendency to attribute negative characteristics or intent to an external object without unequivocal evidence. Therefore, from a psychodynamic perspective, it can be hypothesized that psychic processes of personification and protagonization of illness are strongly intertwined with depression in chronic diseases (Schattner et al., 2008; Shahar and Lerman, 2013). Indeed, people with diabetes may perceive their chronic illness as a sort of “bad” persecutory object (Marchini et al., 2018), ascribing human attributes to their stressful condition as a separate entity (Shahar and Lerman, 2013). This is confirmed by the association between Projection and lower physical well-being found in the present study. Indeed, diabetes-related limitations and poorer physical status may intensify the perceived illness intrusiveness and the consequent tendency to rely on Projection, thus externalizing the responsibility of care management (D’Alberton et al., 2012; Caputo, 2013; Conti et al., 2016; Marchini et al., 2018). Besides, as Projection involves the justification of aggression against external sources of frustration, such a defense mechanism might lead to the justifying of covert hostile and counter dependent behaviors, such as a bad lifestyle and reduced treatment adherence, in turn negatively affecting one’s physical status. This is in line with previous findings regarding the high levels of frustration and anger in patients with T2DM, mainly due to restrictions on food and comorbidities in sexual life (Zurita-Cruz et al., 2018; Al Anazi et al., 2019).

Another interesting finding refers to Principalization, which is associated with lower depression and better QoL in both physical and mental components. This is consistent with the tendency to repress negative affect, which is typical of Principalization, overall resulting in the improvement of personal well-being. In this regard, several interpretations can be made. On the one hand, Principalization may work as a protective factor against experiencing diabetes distress, thus enacting more rational explanations for such a chronic condition and more effective strategies to handle it without feeling powerless and overwhelmed. In this sense, according to the theoretical framework, it would contribute to the modulation of the experience and the expression of anger, thus reducing negative affect reactivity to daily stressors (McIntyre et al., 2019; Vicario et al., 2019). However, on the other hand, as Principalization involves the splitting of thought content from affect, it could lead to unresolved or denied depression that may have a negative impact in chronic diseases in the long run (Hyphantis et al., 2005).

Aside from this, some conflicting findings emerge with regards to TAS, as such a mechanism correlates positively with physical well-being and negatively with mental well-being. Despite their seeming counterintuitive associations, the sense of guilt can be advocated as a fruitful construct for their understanding. Indeed, the tendency to handle diabetes-related conflicts by directing aggressiveness toward oneself may trigger self-punishment thoughts referring to a persistent sense of self-defectiveness, thus negatively affecting mental well-being. At the same time, self-blame for having symbolically damaged one’s health status may increase the effort in reparation, for example by adopting the correct lifestyle behaviors, monitoring blood glucose and complying with a medical regimen, which contribute to the improvement of one’s physical health status (Marchini et al., 2018).

Furthermore, Reversal, including defenses that act to minimize the severity of perceived threats by responding neutrally or positively toward a frustrating object, was found to be associated with lower depression and higher mental well-being. This result is not surprising, as denial-related defenses aimed at lessening one’s sense of loss are demonstrated to contribute to diabetes adaption to some extent (Marchini et al., 2018), as a way of contrasting the process of somatic disruption (Caputo, 2019a). Several authors have stated that denial may serve as an emotion-focused coping strategy, allowing the reduction of distress perception in patients with diabetes in the short term (Gois et al., 2012; Tripathy et al., 2012; Marchini et al., 2018). However, it should be noted that such a defensive strategy does not necessarily lead to effective self-care behaviors (Hyphantis et al., 2005; Marchini et al., 2018; Caputo, 2019b), as also suggested by the lack of association with physical well-being in the present study.

The findings regarding the potential correlations between defense mechanisms and both time since diagnosis and metabolic control show no significant associations, with the exception of a positive relationship between Projection and time since diagnosis. This result seems to provide further confirmation about the processes of personification and protagonization of diabetes, as the worsening of such a chronic condition over time is intertwined with the increase of aggression toward an external object that is attributed with negative characteristics (Schattner et al., 2008; Shahar and Lerman, 2013; Marchini et al., 2018).

The added value of the current study is the exploration of defense mechanisms as less conscious variables that could potentially affect personal adjustment to a chronic illness, as in the case of T2DM, that are scarcely investigated in the clinical psychological literature. Another strength is the use of a diagnostic interview, in addition to self-report measures, which can be considered as a gold standard, conferring more robustness to the surveys carried out.

However, some study limitations should be acknowledged, such as its cross-sectional nature, the small sample size and the consequent reduced generalizability of the findings. As potential causal relations among the examined variables cannot be inferred, future research could assess the impact of the considered defensive mechanisms in longitudinal studies, including participants with T2DM. Moreover, the results seem to be very promising, based on the size of some correlational index, even if the high correlations among the DMI’s scales could need more investigation in regard to the structural power of this instrument. Aside from this, the inspection of other potential unobserved variables or confounders affecting the detected relations could be proposed, as well as the inclusion of larger samples allowing sub-group analysis for the observation of specific differences (i.e. gender).

## Conclusion

The present study suggests the potential relevance of defense strategies for the understanding of adaptation to chronic illness in patients with T2DM. Specifically, the study findings highlight that such a clinical sample is featured by a higher tendency to rely on defense mechanisms, probably due to the emotional suffering related to illness, as also confirmed by mild depressive symptoms and worse physical and mental well-being compared to Italian normative samples. The detected correlations between defense mechanisms and both depression and health-related QoL highlight the potential presence of processes of personification and protagonization of diabetes, which may increase over time due to the illness intrusiveness and worsening of diabetes symptoms. Similarly, the positive association found between some defensive strategies (especially, Principalization and Reversal) and well-being measures should be cautiously considered because, despite reducing depressive feelings, they do not necessarily affect self-care and better medical adherence. These data could be expedient to project psychological strategies, specifically designed according to everyone’s defense mechanisms and focused on psychological health concerns. Such psychological trajectories may support people living with T2DM, aiming at healthier self-management and at reducing both psychological and physical outcomes.

## Data Availability Statement

The raw data supporting the conclusions of this article will be made available by the authors, without undue reservation, to any qualified researcher.

## Ethics Statement

The study was approved by the Institutional Ethical Committee of the University Hospital “Gaetano Martino,” University of Messina, Messina, Italy. Participants provided their written informed consent to participate in the study.

## Author Contributions

GM made significant contributions to the design of the research study, drafting of the manuscript, performing of the statistical analysis, interpretation of the data, and revision of the manuscript. AC performed the statistical analysis, provided the interpretation of data, and gave significant contribution to the draft part of the manuscript. FB provided substantial contribution in the drafting part of the manuscript. MQ critically revised the manuscript. CV critically revised the manuscript and gave the final approval of the manuscript to be submitted.

## Conflict of Interest

The authors declare that the research was conducted in the absence of any commercial or financial relationships that could be construed as a potential conflict of interest.
